# Preferences of Patients and Providers in High-Burden Malaria Settings for Long-Acting Malaria Chemoprevention

**DOI:** 10.4269/ajtmh.23-0245

**Published:** 2023-08-21

**Authors:** Kimberly K. Scarsi, Harlan Sayles, Kelvin Kapungu, Peter Sifuna, Matthew M. Ippolito, Renae Furl, Matthew J. Anderson, Joelle Dountio Ofimboudem, Gershom Chongwe, Jack Hutter, Steven P. Rannard, Andrew Owen, Susan Swindells

**Affiliations:** ^1^ College of Pharmacy, University of Nebraska Medical Center, Omaha, Nebraska;; ^2^ College of Medicine, University of Nebraska Medical Center, Omaha, Nebraska;; ^3^ College of Public Health, University of Nebraska Medical Center, Omaha, Nebraska;; ^4^ Department of Public Health and Epidemiology, Tropical Diseases Research Centre, Ndola, Zambia;; ^5^ Kombewa Clinical Research Center, Kenya Medical Research Institute (KEMRI)/U.S. Army Medical Research Directorate–Africa (USAMRD-A), Kisumu, Kenya;; ^6^ Department of Medicine, Johns Hopkins University School of Medicine, Baltimore, Maryland;; ^7^ The Johns Hopkins Malaria Research Institute, John Hopkins Bloomberg School of Public Health, Baltimore, Maryland;; ^8^ Treatment Action Group, New York, New York;; ^9^ Tropical Diseases Research Centre, Ndola, Zambia;; ^10^ Department of Chemistry, Centre of Excellence in Long-Acting Therapeutics (CELT), University of Liverpool, Liverpool, United Kingdom;; ^11^ Department of Molecular and Clinical Pharmacology, Centre of Excellence in Long-acting Therapeutics (CELT), University of Liverpool, Liverpool, UK

## Abstract

Antimalarial medications are recommended for chemoprevention as part of malaria control programs to decrease the morbidity and mortality related to more than 200 million infections each year. We sought to evaluate patient and provider acceptability of malaria chemoprevention in a long-acting formulation. We administered questionnaires to patients and providers in malaria endemic districts in Kenya and Zambia. Questions explored preferences and concerns around long-acting antimalarial formulations compared with oral formulations. We recruited 202 patient respondents (Kenya, *n =* 102; Zambia, *n =* 100) and 215 provider respondents (Kenya, *n =* 105; Zambia, *n =* 110). Long-acting injection was preferred to oral pills, whereas oral pills were preferred to implant or transdermal administration by patient respondents. Of 202 patient respondents, 80% indicated that they ‘definitely would try’ malaria chemoprevention offered by injection instead of oral pills. Of parents or guardians, 84% of 113 responded that they ‘definitely would’ have their child age < 12 years and 90% of 88 ‘definitely would’ have their child ≥12 years receive an injection for malaria prevention. Provider respondents indicated that they would be more likely to prescribe a long-acting injectable product compared with an oral product for malaria chemoprevention in adults (70%), adolescents ages 12 years and older (67%), and children <12 years (81%). Potential for prolonged adverse effects with long-acting products was the highest concern for patient respondents, while higher medication-related cost was cited as the most concerning barrier to implementation by providers. Overall, these findings indicate enthusiasm for the development of long-acting injectable antimalarials to provide individual delivery method options across age groups.

## INTRODUCTION

Malaria, caused by protozoan parasites of the *Plasmodium* genus and transmitted by anopheline mosquitoes, remains entrenched across sub-Saharan Africa where > 95% of the global burden is borne.^1^ In 2021, there were an estimated 247 million malaria cases and 619,000 malaria-related deaths.^1^ Comprehensive malaria programs include both malaria eradication and measures to reduce morbidity and mortality.^2^ In contrast to every other inhabited continent, no sub-Saharan African nation has successfully eliminated malaria. Since 2015, malaria has resurged in some parts of the subcontinent despite unprecedented investments in malaria control over the first 2 decades of the millennium,^3^ and the recent incursion of the species *Anopheles stephensi*, an efficient malaria vector in urban settings, into the Horn of Africa threatens to further destabilize malaria control efforts.^4^

The concept of a “chemical vaccine” for malaria^5^ has been proposed as an important addition to the antimalarial armamentarium in the current era of arrested progress.^1^ The concept, which refers to long-acting pharmaceuticals for prevention, is especially timely: in its latest revision, WHO guidelines greatly expand the potential applications of pharmacologic-based control measures.^1^ Perennial malaria chemoprevention, intermittent preventative treatment of malaria in school-age children, post-discharge malaria chemoprevention after hospitalization, and seasonal malaria chemoprevention are recommended as prevention strategies to decrease malaria burden and malaria-associated adverse outcomes for children living in areas of moderate to high malaria transmission.^6^ In adults, the WHO recommends antimalarials for the intermittent preventive treatment of malaria during pregnancy and, in a narrow range of circumstances, for mass drug administration.

Long-acting formulations of medications are available for the treatment or prevention of several conditions, including mental health, contraception, and HIV prevention.^7^^–^^11^ Specific to prevention strategies, long-acting formulations result in higher rates of prevention compared with oral options, potentially related to user preference and convenience leading to higher rates of medication adherence.^9^ The relative ease of production and distribution along with the strain-transcendence of long-acting formulations of antimalarial drugs, vis-à-vis the RTS,S/AS01 and R21 vaccines,^12^^,^^13^ contributes to their potential attractiveness for malaria chemoprevention. The most recent update to the WHO guidelines for malaria includes greater provision and latitude than any prior edition for national control programs to deploy chemotherapeutics for control and elimination,^6^ and long-acting formulations may simplify implementation, reduce medication nonadherence, and extend the duration of efficacy.

The growing use of long-acting medications has identified important patient preferences that influence the uptake of, and adherence to, medication products. Further, clinician and policymaker opinions will drive the inclusion of long-acting therapies into guidelines and practice. To inform long-acting formulation development and deployment, we conducted a survey of Kenyan and Zambian patient end-users, prescribers, and policymakers to evaluate the acceptance of and potential concerns about long-acting formulations of malaria chemoprevention.

## MATERIALS AND METHODS

**Figure 1.**
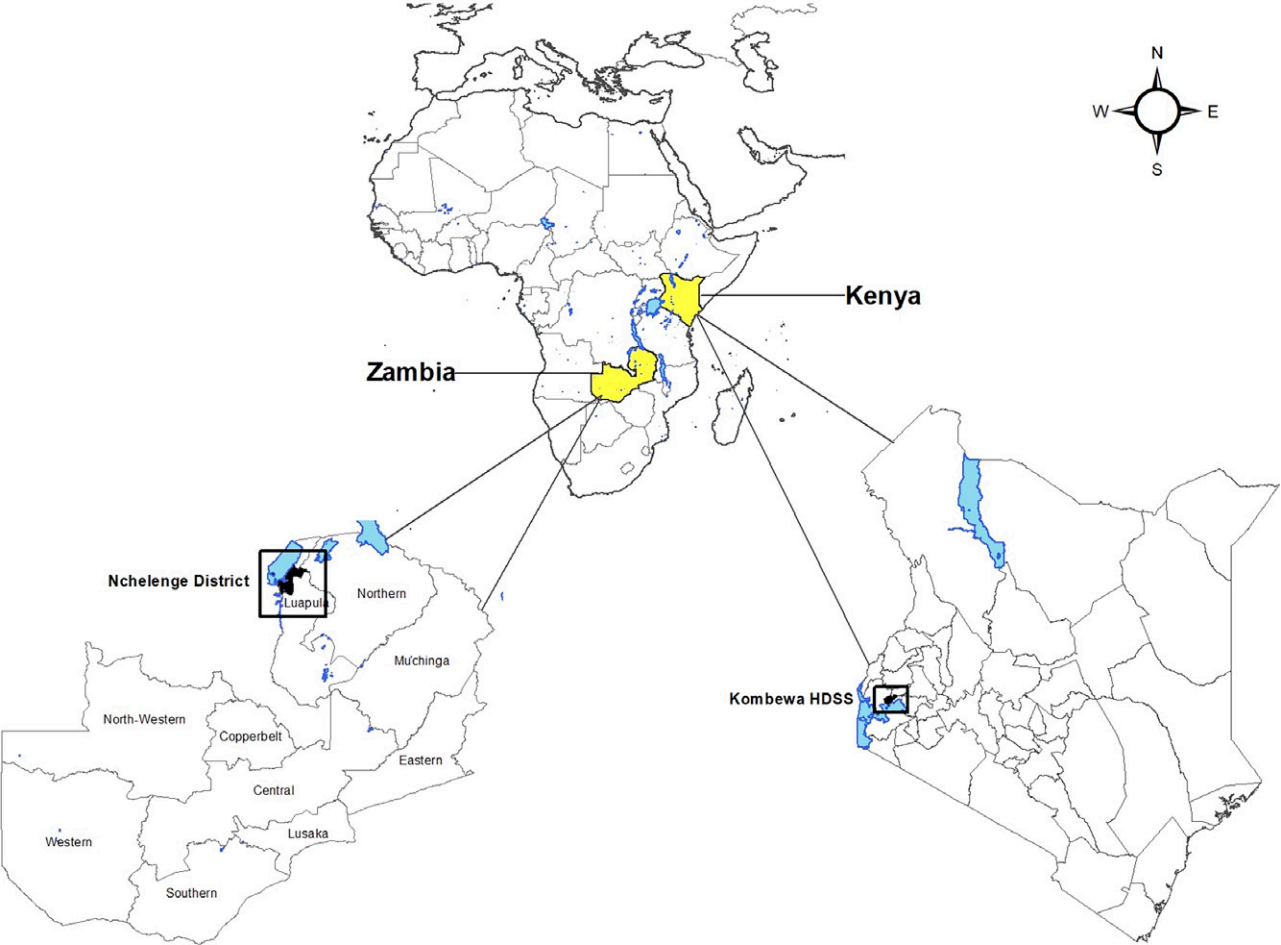
Regions of survey implementation.

We conducted a descriptive, cross-sectional survey of patients, healthcare workers, and public health officials living in malaria endemic areas in Kenya and Zambia (Figure 1). Individuals who were not medical or public health officials are referred to as “patients” herein. They were not required to currently have malaria but represent the target end-user of a future malaria chemoprevention strategy. In Kenya, a population-based surveillance platform established in a holoendemic malaria zone in western Kenya was used to identify and locate potential survey respondents. A listing of patient respondents was generated from the residency database and shared with the field team for purposes of scheduling and administering questionnaires. Provider respondents were identified from health facilities with varying levels of care within the study area. In Zambia, purposive sampling was used to identify stakeholders and a district where malaria was highly prevalent. Five health facilities participated in the Nchelenge district of Zambia: Nchelenge Primary Health Center (PHC), Kashikishi PHC, Kafutuma PHC, Kabuta PHC, and Saint Paul’s General Hospital. Convenience sampling was used at the health facilities to recruit patients and providers. Patient respondents may have been attending the health facility for any purpose, including routine preventative health visits. Institutional review boards at all sites approved the study and all respondents provided verbal (Kenya) or written (Zambia) informed consent.

### Study methods and data collection.

Study data were collected and managed using Research Electronic Data Capture (REDCap) electronic data capture tools hosted at the University of Nebraska Medical Center (https://projectredcap.org/resources/citations/). Questionnaires with closed-ended questions were used to collect deidentified information about patient or provider demographics, attitudes to, interest in, and potential concerns about long-acting formulations for malaria chemoprevention (surveys available in Supplemental Material). The questionnaire for patients also had questions on current medication practices and adherence behaviors, experience with injectable medications, malaria history, preferred methods of malaria prevention, perceived risks and/or benefits of getting an injection, and willingness to adopt a new method for malaria prevention. A similar questionnaire for providers had additional questions on work environment, professional training, willingness to use or prescribe long-acting malaria chemoprevention agents, and potential organizational barriers to their use. The patient survey included questions related to long-acting delivery via intramuscular or subcutaneous injection, subdermal implant, or transdermal patch, whereas the provider survey focused on injectable options. Both surveys asked respondents to consider responses in contrast to locally available oral options but did not describe effectiveness of a new product.

Pilot survey data and survey administrator feedback were used to improve reliability, ensure proper content, and optimize consistent survey administration. Trained survey administrators conducted in-person interviews with patients and providers and recorded responses in REDCap using electronic tablets. If needed, the survey administrators translated the English survey into preferred local languages (Bemba, Kiswahili, or Dholuo).

### Statistical methods.

Responses were summarized using means and standard deviations for continuous measures such as participant age and preference rankings and using counts and percentages for binary and categorical measures. Comparisons of continuous measures between two groups were evaluated using *t* tests, whereas comparisons of categorical measures between two or more groups were evaluated using Pearson’s χ^2^ tests or Fisher’s exact tests as indicated. Differences in responses were explored based on country, sex, age, current oral medication use, recent adherence to current oral medication, and prior injectable medication use. Analyses were done using Stata v17 (StataCorp LLC, College Station, TX).

## RESULTS

### Patient survey results.

We enrolled 202 patient respondents. Table 1 describes the participant demographics and clinical characteristics. The patient respondents had a mean age of 37 (range: 18–90) years and reported a similar distribution of sex in each country. Prior receipt of injectable medication use was common at both sites but was higher in Zambia than Kenya. Of the 32% of respondents taking daily oral medications for any indication, 45% missed a dose of oral medication within the past week, and an additional 25% missed a dose in the past 1 to 2 weeks. Nearly all respondents (97%) reported having malaria in the past.

**Table 1:** Patient participant demographics and clinical characteristics

Characteristics	Overall (*N* = 202)	Kenya (*n* = 102)	Zambia (*n* = 100)	*P* value*
Race/ethnicity, *n* (%)				
Black	202 (100)	102 (100)	100 (100)	–
Age in years, mean (range)	37 (18–90)	40 (18–90)	35 (19–71)	0.035†
Age groups, *n* (%)				
18–24	50 (25)	21 (21)	29 (29)	
25–34	51 (25)	28 (27)	23 (23)	0.491
35–44	45 (22)	22 (22)	23 (23)	
45+	56 (28)	31 (30)	25 (25)	
Sex, *n* (%)				
Male	97 (48)	48 (47)	49 (49)	0.78
Female	105 (52)	54 (53)	51 (51)	
Currently pregnant, *n* (%)				
Yes	10 (10)	5 (9)	5 (10)	0.87‡
No	94 (90)	49 (91)	45 (88)	
Don’t know	1 (1)	0 (0)	1 (2)	
Currently breastfeeding, *n* (%)				
Yes	22 (21)	10 (19)	12 (24)	0.56
No	82 (78)	43 (81)	39 (76)	
Currently taking any medication in pill form, *n* (%)				
Yes	64 (32)	36 (35)	28 (28)	0.27
No	138 (68)	66 (65)	72 (72)	
Number of pills per day, *n* (%)				
0 or < 1	8 (13)	7 (19)	1 (4)	0.068‡
1–2	45 (70)	25 (69)	20 (71)	
3–5	8 (13)	4 (11)	4 (14)	
6–9	3 (5)	0 (0)	3 (11)	
Last reported missed dose, *n* (%)				
Within the past week	29 (45)	17 (47)	12 (43)	0.877‡
1–2 weeks ago	16 (25)	10 (28)	6 (21)	
3–4 weeks ago	6 (9)	3 (8)	3 (11)	
1–3 months ago	3 (5)	1 (3)	2 (7)	
More than 3 months ago	10 (16)	5 (14)	5 (18)	
Received medications by injection, *n* (%)				
Yes	159 (79)	71 (70)	88 (88)	0.001‡
No	41 (20)	30 (30)	11 (11)	
Unsure or don’t know	1 (1)	0 (0)	1 (1)	
Prior malaria infection, *n* (%)				
Yes	195 (97)	97 (96)	98 (98)	0.56‡
No	4 (2)	2 (2)	2 (2)	
Unsure or don’t know	2 (1)	2 (2)	0 (0)	

* Comparisons between Zambia and Kenya respondents, *P* values from Pearson χ^2^ test unless indicated.

† Student’s *t* test.

‡ Fisher’s exact test.

**Figure 2.**
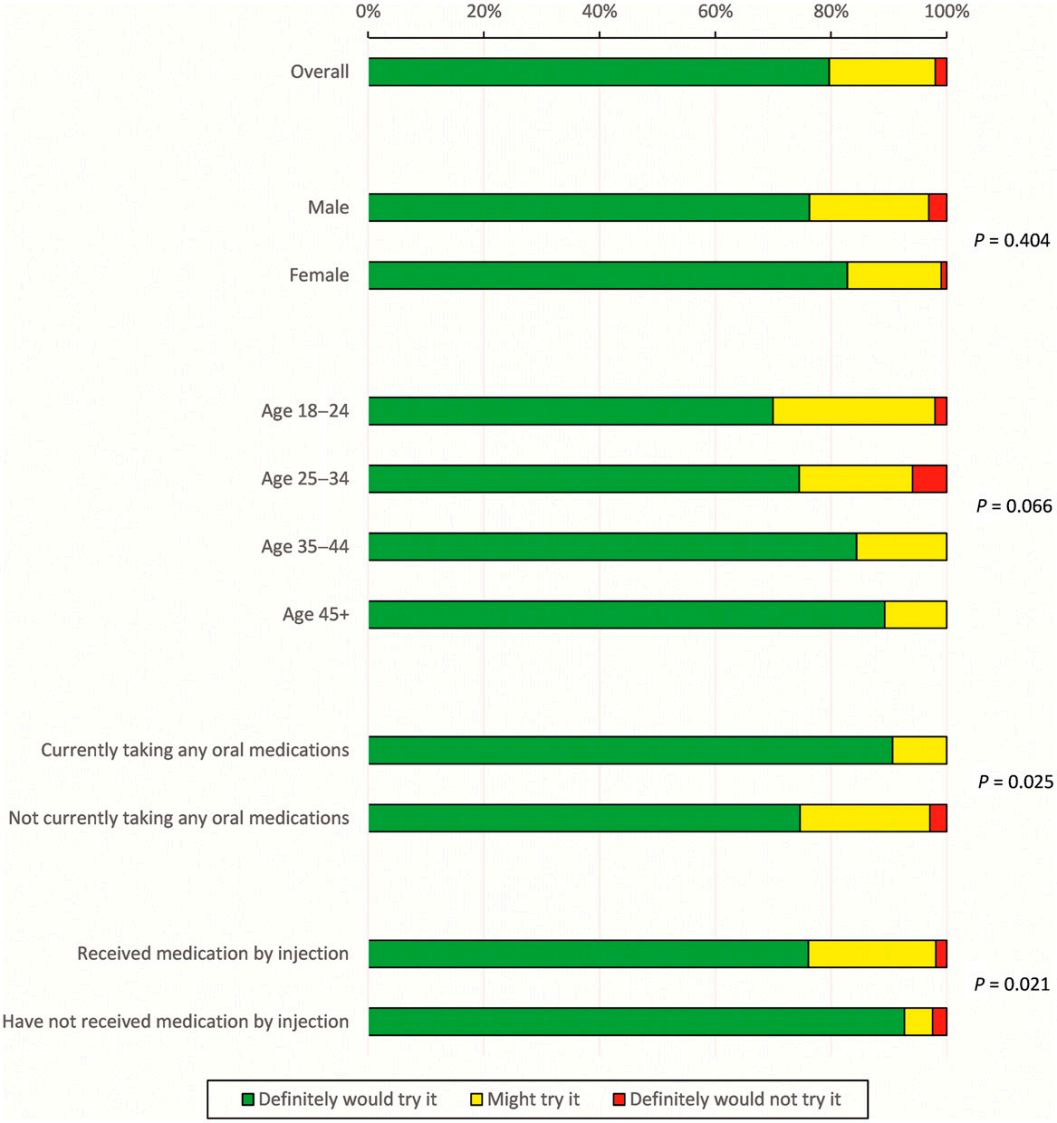
Patient responses to whether they would take a long-acting injectable product instead of an oral product for malaria chemoprevention. Results are reported overall as well as by demographic or clinical characteristics. *P* values are from Fisher’s exact tests.

Most patient respondents indicated that they would try malaria chemoprevention offered by injection instead of oral options (80% “definitely would try it,” 18% “might try it,” 2% “definitely would not try it”; Figure 2). No difference in interest was observed based on sex, age, or recent adherence to oral medications. Interest in injectables was higher among those who were currently taking oral pills compared with those not receiving oral pills (“definitely would try it”: 91% versus 75%, *P* = 0.025). Individuals who reported prior use of injectable medications had less interest in long-acting injectable chemoprevention compared with those who did not report prior use (“definitely would try it”: 76% versus 93%, *P* = 0.021).

**Figure 3.**
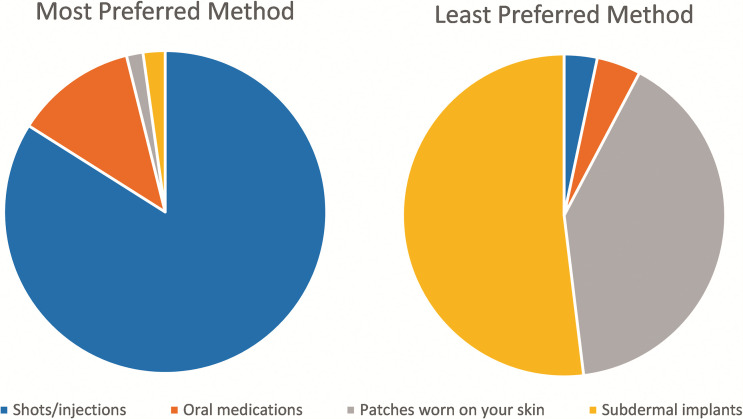
Preferences of patients for most and least preferred method of malaria chemoprevention administration.

When asked to rank their preferred route of administration for medications, injection was the first choice for 84% of patient respondents and the second choice for an additional 10% (Figure 3). Oral formulations were the first choice for 12% and the second choice for 75%. Implants and patches were least preferred overall, being the top choice for 2% each. Implants were least preferred among males (last choice for 69%), and patches were least preferred among females (last choice for 53%). Patients perceived injections to be more effective than other routes of administration: 81% described injections as “strong/very effective,” compared with 38% for oral agents, 20% for implants, and 5% for patches.

There was some variability in reported enthusiasm for long-acting injectables based on the frequency of clinic visits required: 52% “definitely would” try a monthly injection, 41% every 2-month injection, and 55% every 3-month injection. More respondents in Zambia preferred monthly injections compared with Kenya (86% versus 39%, *P* < 0.001), whereas more respondents in Kenya preferred every-3-month injection compared with Zambia (76% versus 66%, *P* = 0.011). Self-administration of subcutaneous injections was not highly desirable to patient respondents: 38% indicated they “definitely would give it to myself,” and 32% said they “definitely would not give it to myself.”

**Figure 4.**
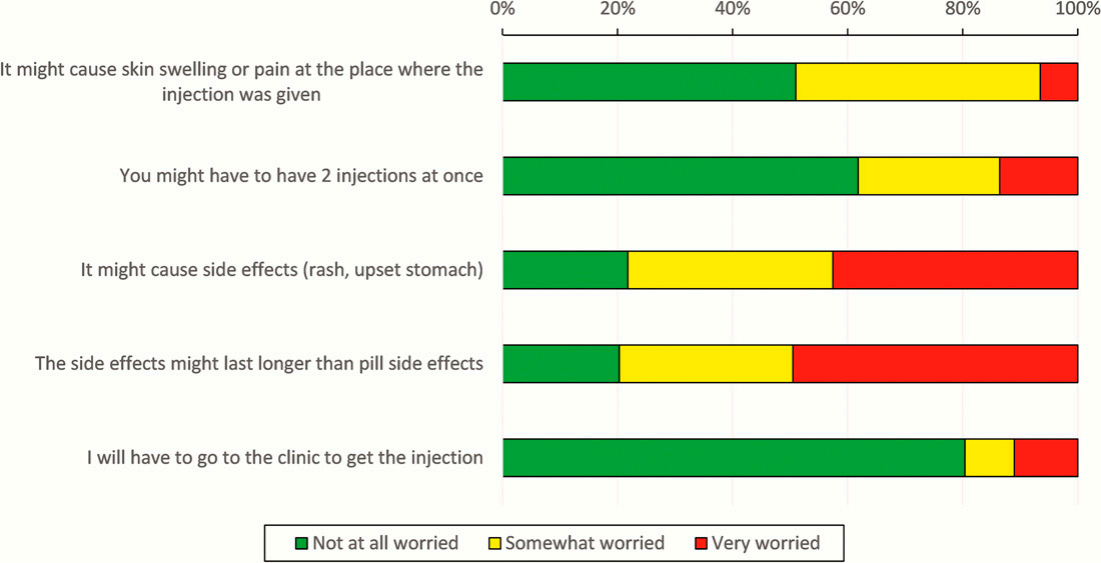
Patient reported concerns with injectable long-acting malaria chemoprevention.

Respondents indicated that improved effectiveness (87%), fewer side effects (76%), ease of administration (85%), and discretion (75%) were all “very beneficial” attributes of an injectable medication. The most common concern about long-acting injectable medications among patient respondents was that side effects may last longer compared with oral medications (Figure 4). This concern was more common among females (*P* = 0.041), those who previously received injections (*P* = 0.012), and those who did not currently take oral medications (*P* < 0.001).

Of patient respondents who were a parent or guardian of a child under 12 years, 95 of 113 (84%) responded that they “definitely would” have their child receive an injection for malaria prevention. Thirty-five percent reported being “very worried” about side effects, and 56% were “very worried” about side effects lasting longer (Table 2). Of parents and guardians of children 12 years or older, 79 of 88 (90%) “definitely would” have their child receive an injection for malaria prevention, with 47% indicating they were “very worried” about the duration of side effects being longer (Table 2).

**Table 2:** Parent and guardian participant concerns about long-acting injectable malaria chemoprevention for their children

Concerns	Not at all worried, *n* (%)	Somewhat worried, *n* (%)	Very worried, *n* (%)
Concerns for children < 12 years (*N* = 115)			
Receipt of injection	92 (81)	18 (16)	4 (4)
Pain with injection	60 (53)	42 (37)	11 (10)
More clinic visits	83 (78)	6 (6)	18 (17)
Cause side effects	25 (22)	49 (43)	40 (35)
Longer duration of side effects compared with pills	20 (18)	30 (26)	64 (56)
Concerns for children ≥ 12 years (*N* = 88)			
Receipt of injection	72 (84)	11 (13)	3 (3)
Pain with injection	47 (53)	39 (44)	2 (2)
More clinic visits	77 (89)	6 (7)	4 (5)
Cause side effects	22 (25)	35 (40)	31 (35)
Longer duration of side effects compared with pills	15 (17)	32 (36)	41 (47)

### Provider survey results.

**Figure 5.**
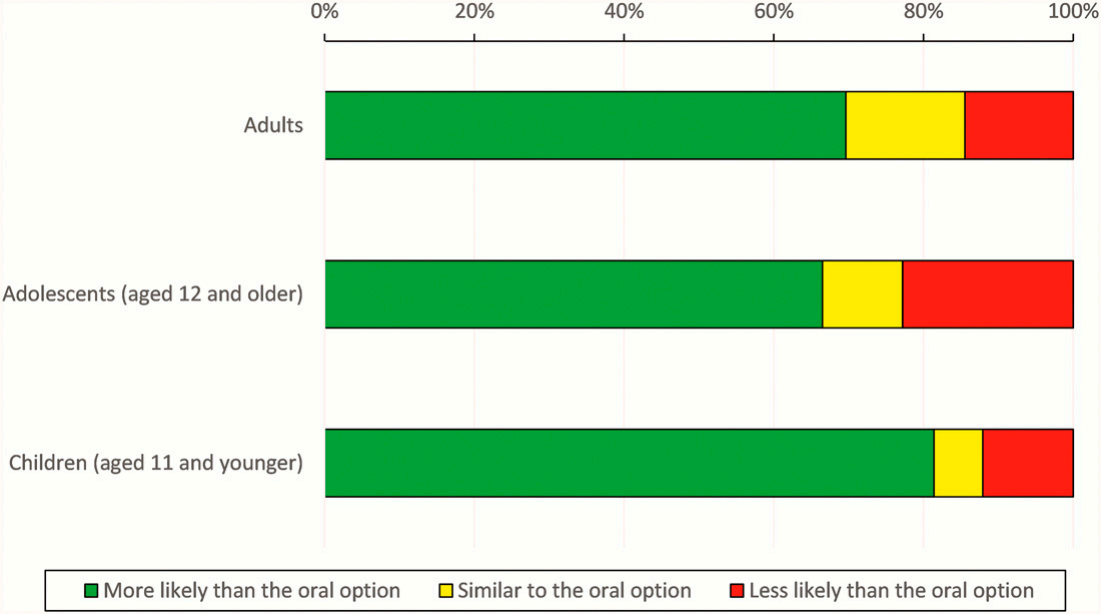
Provider responses to whether they would prescribe a long-acting injectable product instead of an oral product for malaria chemoprevention.

We enrolled 215 providers (Table 3). Most provider respondents were nurses (61%), working at ministry of health or health department clinical sites (61%), and 80% had worked in a malaria test-and-treat program. Most were current prescribers (90%), of whom 55% reported prescribing malaria chemoprevention for > 100 patients in the past year. Overall, provider respondents indicated that they would be more likely to prescribe a long-acting injectable product compared with an oral product for malaria chemoprevention in adults (70%), adolescents aged 12 years and older (67%), and children < 12 years (81%) (Figure 5). No difference in interest was observed based on professional background; however, those with experience in reactive test-and-treat programs had a higher interest in long-acting options than oral pills (“definitely would try it”: 74% versus 51%, *P* < 0.001).

**Table 3:** Provider participant demographics

Characteristics	Overall (*N* = 215)	Kenya (*n* = 105)	Zambia (*n* = 110)	*P* value*
Sex, *n* (%)				
Male	112 (52)	51 (49)	61 (55)	0.31
Female	103 (48)	54 (51)	49 (45)	
Years in practice, *n* (%)				
0–5	100 (47)	33 (31)	67 (61)	< 0.001
6–10	62 (29)	38 (36)	24 (22)	
11–15	25 (12)	18 (17)	7 (6)	
16–20	13 (6)	9 (9)	4 (4)	
21 or more	15 (7)	7 (7)	8 (7)	
Professional background, *n* (%)				
Medical doctor or medical officer	14 (7)	4 (4)	10 (9)	0.043
Nurse	132 (61)	60 (57)	72 (65)	
Clinical officer or medical licentiate	46 (21)	28 (27)	18 (16)	
Pharmacist/other	19 (9)/4 (2)	9 (9)/4 (4)	10 (9)/0 (0)	
Practice description, *n* (%)				
Private office or clinic	18 (8)	18 (17)	0 (0)	< 0.001
Community hospital/clinic	41 (19)	16 (15)	25 (23)	
Local or regional public health clinic	24 (11)	3 (3)	21 (19)	
Health department or ministry of health	132 (61)	68 (65)	64 (58)	
Number of patients they prescribed malaria chemoprevention in the past year, *n* (%)				
< 10	31 (16)	2 (2)	29 (30)	< 0.001
11–100	55 (29)	23 (25)	32 (33)	
101–500	35 (18)	17 (18)	18 (18)	
> 500	69 (36)	50 (54)	19 (19)	

* Comparisons between Zambia and Kenya respondents, *P* values from Fisher’s exact tests.

Provider respondents supported long-acting injectable therapy irrespective of frequency of injection, with the highest interest when administered every 3 months (71%), followed by monthly administration (63%) and every 2-month injections (51%). Regarding where and how injections should take place, most providers preferred that injections were administered during visits to a clinical site (88% first choice). Only 10% indicated that having the injection done by a community healthcare worker would be their first choice, and 2% indicated home administration of an injection by a family member as first choice. The deltoid was ranked the most preferred site for an intramuscular injection by 65% of providers and was the second choice for another 18%. The gluteal muscle was the preferred choice for 30% of providers and the second choice for an additional 32%. The thigh was the preferred choice for only 3% of providers, and 41% listed it as their second choice. Subcutaneous administration was least preferred, with a large majority listing it as their last (69%) or next to last (20%) choice.

**Figure 6.**
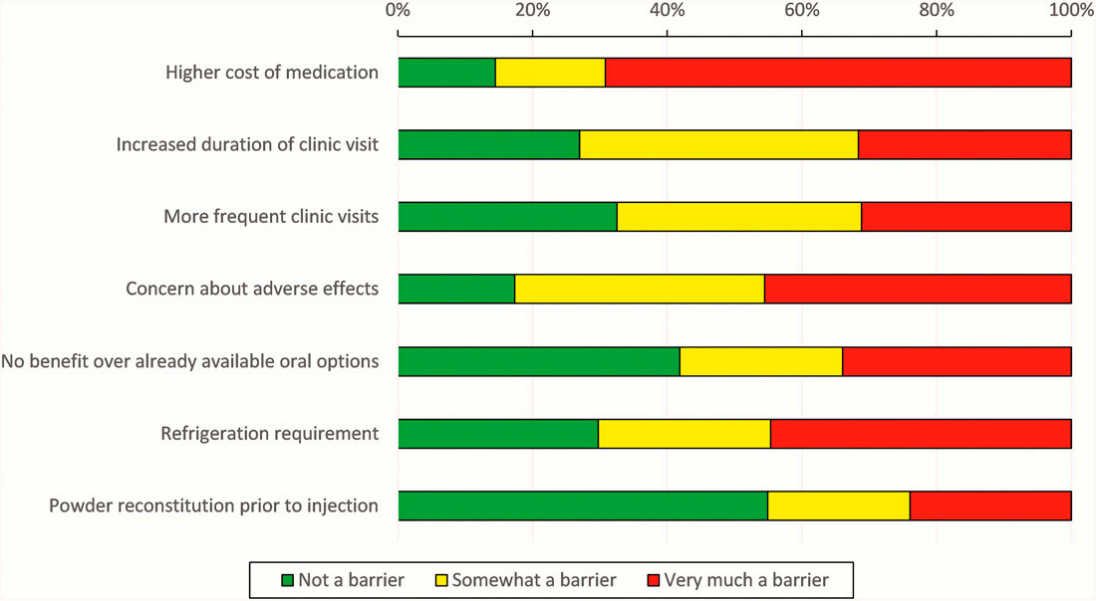
Provider perceived barriers with injectable long-acting malaria chemoprevention.

Providers rated all proposed benefits associated with long-acting injectable products highly. Improved public health associated with long-term malaria prevention was the most cited “very beneficial” attribute of long-acting therapy (90%). Potentially higher cost was rated as the highest barrier to implementation of a long-acting injectable products (69%), with concern about adverse events the second highest barrier (46%) (Figure 6).

## DISCUSSION

We conducted a research survey of patients and providers to understand preferences around long-acting malaria chemoprevention. Respondents in both groups indicated high levels of enthusiasm for long-acting injectable formulations for both adults and children. Patient respondents currently taking oral pills had a significantly higher level of interest in long-acting injections, whereas those who had previously received an injection had lower interest. For provider respondents, those who previously participated in a reactive test-and-treat program had a higher level of interest. All respondents were enthusiastic about potential personal and health-system benefits associated with a long-acting product, including increased adherence, prolonged duration of effect, and ease of administration. Patient respondents were most concerned about prolonged adverse effects related to long-acting agents compared with conventional oral agents. Provider respondents were particularly concerned about increased costs associated with long-acting formulations. Among patient respondents receiving daily oral therapy for any indication, more than half reported missing a dose in the prior 2 weeks, representing an opportunity for improved medication adherence with long-acting therapy. To our knowledge, this is the first survey of patients or providers regarding long-acting malaria chemoprevention. The similarities between the provider and patient responses are reassuring and offer useful insight for both product development and areas where additional education of end-users may be necessary to increase uptake of a novel long-acting product.

We observed interesting findings that require consideration when developing or implementing a long-acting medication. First, respondents who had previously received injectable medications had a lower interest in long-acting injectable therapy. This may be related to prior adverse events experienced with an injection, fear of needles, or other barriers to accessing or receiving injections. Second, although interest in a long-acting injectable chemoprevention remained high irrespective of the dosing interval, we noted a difference between countries. Kenyan respondents preferred less frequent dosing (every 3 months). We expected this preference, considering less transportation costs or time away from personal or work responsibilities. However, in Zambia, both patients and providers preferred a monthly injection over longer dosing intervals. Although not specifically asked in the survey, survey administrators and local investigators described a concern that a less frequent injection would not work as well or that patients may forget to return to clinic if the interval was greater than 1 month. Patient respondents also reported a desire to see the provider more frequently through monthly injections. Finally, oral therapy was preferred by patient respondents compared with long-acting implant or transdermal administration, and neither patients nor providers preferred self-administration of a subcutaneous injection. These results may be due to less experience with these modes of administration. For recently approved long-acting antiretroviral medicines, the understanding of patient, provider, and policymaker preferences has proven to be essential for targeting clinical trials to appropriate populations and subgroups, as well as for targeting deployment.^14^^–^^17^ Much of the early preference research in HIV was conducted in high-income countries, largely did not include providers, and research to understand interests and attitudes toward long-acting formulations in low- and middle-income countries (LMICs) has been slow to emerge. For malaria, the greatest impact opportunity for novel drug-delivery technologies is among individuals with the greatest burden of disease, who overwhelmingly reside in LMICs. Thus, prioritizing preference research in these LMICs will be critical to inform development and downstream deployment of emerging long-acting technologies.

Outside of vaccines, current efforts to develop long-acting chemoprevention interventions for malaria are focused either on the application of advanced formulation technologies to repurpose existing small molecule drugs,^18^ or through development of monoclonal antibodies, which possess inherently long elimination half-lives that may be further extended by Fc modification.^19^ For small molecule drugs, the predominant focus has been on the development of injectable formulations analogous to those recently approved for HIV treatment and prevention, which provide long periods of drug exposure by reducing the absorption rate to extend the half-life. This has included the development of novel drugs optimized for low solubility and high metabolic stability,^20^ but new drug development and approval can take more than a decade for products to reach patients, if they do at all. The repurposing of an existing drug from an oral to an injectable format faces fewer regulatory barriers because the existing drug safety is already well understood. Accordingly, the already-approved oral drug atovaquone has attracted recent interest as a potential candidate for chemoprevention because of its inherent low solubility and low clearance.

Two efforts are underway to develop atovaquone as a long-acting injectable. The first involves the application of nanotechnologies to the native drug to produce an injectable format able to provide long periods of pharmacokinetic exposure and protection from malaria acquisition in established animal model systems.^21^ This approach is the focus of our work and is being developed with funding from Unitaid in project LONGEVITY (https://www.liverpool.ac.uk/centre-of-excellence-for-long-acting-therapeutics/longevity). The second approach involves the formulation of a rapidly hydrolysable prodrug of atovaquone, which enables compatibility with other long-acting injectable formulation approaches, being developed by the Medicines for Malaria Venture (https://www.mmv.org/mmv-pipeline-antimalarial-drugs/mmv371).

This study has several strengths and limitations. We believe our respondents were well-representative of the population that may benefit from a long-acting malaria chemoprevention medication: all respondents were from a region of high malaria transmission, nearly all patient respondents had malaria in the past, and provider respondents were experienced in malaria chemoprevention and treatment. However, we did not capture the patient respondents’ prior experience with severe malarial infection for themselves or their children, nor perceived risk of future malaria infections. Second, the survey was only conducted in Kenya and Zambia and may not represent other malaria-endemic countries or geographic regions. Finally, given the diversity of preferred languages, even within each country, the surveys were written in English and verbally translated by the survey administrator. Although we conducted training before implementing the survey, interpretation of the question may not have been consistent across all study staff or survey respondents.

These findings demonstrate high levels of enthusiasm from both patients and providers in a long-acting malaria chemoprevention strategy by injection. Information from these surveys will help focus development activities so that interests and attitudes of patients and healthcare providers are captured early in development and integrated into emerging target product profiles and research activities. However, a novel long-acting chemoprevention will only be impactful if it is widely accessible and affordable in regions of the world with the heaviest burden of malaria. Therefore, cost-efficiency and scalability remain critical considerations in tandem with patient and provider preferences. Building on our growing experience with long-acting medications for other conditions, a long-acting antimalarial stands to offer individualized choice, improved effectiveness, and increased patient adherence, all valuable prevention tools as we work toward malaria eradication.

## Supplemental Materials


Supplemental materials
