# Supplementary material for: Preferences of Patients and Providers in High-Burden Malaria Settings for Long-Acting Malaria Chemoprevention
Source: Am J Trop Med Hyg. 2023 Aug 21;109(4):752–60. doi: 10.4269/ajtmh.23-0245 (PMC10551098; doi:10.4269/ajtmh.23-0245)
Supplement: Supplementary file 1 [file tpmd230245.SD1.pdf]

Supplemental Material:

Preferences of patients and providers in high-burden malaria settings for long-acting malaria chemoprevention

Supplemental Material Index:

Survey administered to Patient participants ………………………………………………………….. Page 2

Survey administered to Provider participants ………………………………………………………… Page 7

LONGEVITY Malaria Patient Survey

Please complete the survey below.

Thank you!

Tablet User Name (do NOT edit) ______________________

1.) This is a list of terms that people use to describe their sex, please check a term that applies to you.

◯ Male
◯ Female
◯ I prefer not to answer

1a.) Are you currently breast feeding?

◯ Yes
◯ No

1b.) To your knowledge, are you now pregnant?

◯ Yes
◯ No
◯ Don't know / Not sure

2.) What is your race/ethnicity, please check all terms that apply to you.

☐ Black African
☐ Hispanic or Latinx (regardless of race)
☐ Multiple races
☐ White (non-Hispanic)
☐ None of these terms apply
☐ I prefer not to answer

2a.) Please describe your race. ______________________

3.) In what year were you born? ______________________
((__ __ __ __))

4.) Are you taking any medication in the pill form now for any reason?

◯ Yes
◯ No

4a.) Approximately how many pills do you take each day?

◯ 0 or < 1
◯ 1-2
◯ 3-5
◯ 6-9
◯ >9

4b.) Many people intend to take their medicine every day but it is challenging to remember to do so, and it is normal to sometimes forget to take a dose. When was the last time you missed any of your medicines?

◯ Within the past week
◯ 1-2 weeks ago
◯ 3-4 weeks ago
◯ 1-3 months ago
◯ More than 3 months ago

5.) Have you ever taken medications by injection instead of taking them by mouth? We are NOT asking about vaccinations. Some examples are: penicillin or antibiotic shots for infections, injections for pain, insulin or birth control like Depo-Provera.

◯ Yes
◯ No
◯ I don't know

6.) Please rate the effectiveness (how strong or how well you think a medicine works) of the following methods of treatment on a scale of 1-3, with 3 being a strong or very effective method of treatment, and 1 being weak or ineffective.

	1-Weak/ineffective	2-Somewhat effective	3-Strong/very effective	Don't know
Under the skin implants*	◯	◯	◯	◯
Patches worn on your skin*	◯	◯	◯	◯
Pills*	◯	◯	◯	◯
Shots/injections*	◯	◯	◯	◯

*Treatment method examples:

a. Under the skin implants

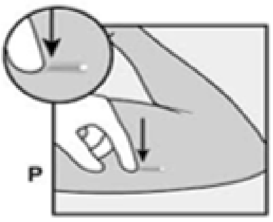

b. External patches on your skin

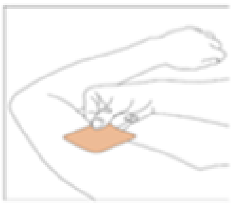

c. Pills

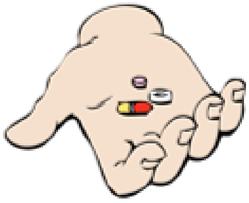

d. Shots/Injections

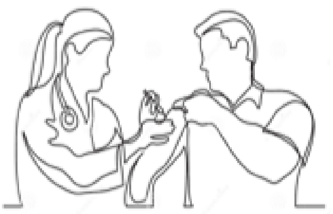

7.) Please rank your preference for ways to take a medicine on a scale of 1-4, with 4 being how you would most prefer to take medicine, and 1 being how you would least prefer to take medicine.

	1-Least prefer	2	3	4-Most prefer
Under the skin implants	◯	◯	◯	◯
Patches worn on your skin	◯	◯	◯	◯
Pills	◯	◯	◯	◯
Injections	◯	◯	◯	◯

8.) Have you ever been told you have malaria or believed that you had malaria?

◯ Yes
◯ No
◯ I don't know

8a.) How many times?

____

9.) Have you ever taken medications to prevent getting malaria?

◯ Yes
◯ No
◯ I don't know

10.) If you were offered malaria prevention by injection instead of oral pills, would you try it?

◯ Definitely would try it
◯ Might try it
◯ Definitely would not try it

11.) When you think about getting an injection for malaria prevention how worried are you that:

	Not at all worried	Somewhat worried	Very worried
It might cause skin swelling or pain at the place where the injection was given	◯	◯	◯
You might have to have 2 injections at once	◯	◯	◯
It might cause side effects (rash, upset stomach)	◯	◯	◯
The side effects might last longer than pill side effects	◯	◯	◯
I will have to go to the clinic to get the injection	◯	◯	◯

12.) When you think about getting an injection for malaria prevention, how beneficial do you consider each of the following things to be?

	Not at all beneficial	Somewhat beneficial	Very beneficial	Don't know
It will work better than pills	◯	◯	◯	◯
It will have less side effects than pills	◯	◯	◯	◯
It will be easier than taking pills	◯	◯	◯	◯
People will not know that I am taking medicine	◯	◯	◯	◯

13.) This new way of taking your medicine may involve injections to prevent malaria. How likely would you be to try this new method if you were required to come to the clinic or doctor's office:

	Definitely would try it	Might try it	Definitely would not try it
...once a month	◯	◯	◯
...every two months	◯	◯	◯
...every three months	◯	◯	◯

14.) Some injections are given under the skin with a small needle that you could be taught to give yourself. If an injection for preventing malaria was given just under the skin with a small needle (like insulin), would you be willing to give it to yourself at home instead of coming to the clinic of doctor's office?

◯ I definitely would give to myself at home
◯ I might give to myself at home
◯ I definitely would not give to myself at home

15.) How likely would you be to try this new method if you were required to give yourself an injection under the skin with a small needle (please mark one box in each row):

	Definitely would try it	Might try it	Definitely would not try it
...once a month	◯	◯	◯
...every two months	◯	◯	◯
...every three months	◯	◯	◯

16.) Are you a parent or guardian of a child who is under 12 years old?

◯ Yes
◯ No

16a.) If the new injection for preventing malaria was available for children younger than 12 years old, would you have your child receive it?

◯ I definitely would have my child receive it
◯ I might have my child receive it
◯ I definitely would not have my child receive it

16b.) When you think about getting an injection for malaria prevention for your child who is under 12 years old, how worried are you that:

	Not at all worried	Somewhat worried	Very worried
They will have to get an injection.	◯	◯	◯
It will be painful.	◯	◯	◯
It might cause side effects (rash, upset stomach).	◯	◯	◯
The side effects might last longer than pill side effects.	◯	◯	◯
I will have to bring them to the clinic to get the injection.	◯	◯	◯

17.) Are you a parent or guardian of a child who is 12 years or older?

◯ Yes
◯ No

17a.) If the new injection for preventing malaria was available for children aged 12 years or older, would you have your child receive it?

◯ I definitely would have my child receive it
◯ I might have my child receive it
◯ I definitely would not have my child receive it

17b.) When you think about getting an injection for malaria prevention for your child who is 12 years or older, how worried are you that:

	Not at all worried	Somewhat worried	Very worried
They will have to get an injection.	◯	◯	◯
It will be painful.	◯	◯	◯
It might cause side effects (rash, upset stomach).	◯	◯	◯
The side effects might last longer than pill side effects.	◯	◯	◯
I will have to bring them to the clinic to get the injection.	◯	◯	◯

18.) Have you ever had to pay for malaria treatment or malaria prevention medicines?

◯ Yes
◯ No

18a.) Think for a moment about how much you currently pay for your malaria treatment, or malaria prevention medicine, or how much you have paid in the past. How likely would you be to try this new way of taking malaria prevention if the cost of this new way of taking medicine is: (Please mark one box in each row).

	Definitely would try it	Might try it	Definitely would not try it
... much less than you pay now	◯	◯	◯
... a little less than you pay now	◯	◯	◯
... the same amount you pay now	◯	◯	◯
... a little more than you pay now	◯	◯	◯
... much more than you pay now	◯	◯	◯

19.) Any other comments (or reasoning if survey is incomplete)?

LONGEVITY Malaria Provider Survey

Please complete the survey below.

Thank you!

Tablet User Name (do NOT edit)

____

1.) What is your sex?

◯ Male
◯ Female
◯ Prefer not to disclose

2.) How many years have you been involved with malaria treatment, prevention, or public health policy?

◯ 0-5
◯ 6-10
◯ 11-15
◯ 16-20
◯ 21 or more

3.) Which of these terms best describes your program/practice? (select one answer)

◯ University medical center/clinic
◯ Private office or clinic
◯ Community hospital/clinic
◯ Local or regional public health clinic
◯ Heath Department / Ministry of Health
◯ Other

3.) Please specify:

____

4.) What is your professional background? (select all that apply)

☐ Medical Doctor or Medical Officer
☐ Nurse (including registered/enrolled/midwives)
☐ Clinical Officer/Medical Licentiate
☐ Pharmacist
☐ Other

4.) Please specify:

____

5.) Have you ever taken part in any of the malaria control or elimination activities listed below? (select all that apply)

☐ Reactive test-and-treat program
☐ Focal test-and-treat program
☐ Other mass drug administration program
☐ None of the above

6.) Are you a clinician who has provided malaria prevention medications to patients within the past year?

◯ Yes
◯ No

6a.) For approximately how many patients have you prescribed malaria prevention medication for in the past year?

◯ < 10
◯ 11-100
◯ 101-500
◯ >500

6b.) What have been the indication(s) for prescribing the malaria prevention medication? (select all that apply)

☐ Intermittent preventive treatment in pregnancy (IPTp)
☐ Intermittent preventive treatment in infants (IPTi)
☐ Intermittent preventive treatment in children (IPTc)
☐ Traveler's prophylaxis
☐ Other
☐ None of the above

6b.) Please specify: __________________________________

6c.) Do you prescribe injectable medications to be administered in your facility, such as antimicrobials, analgesics, or depomedroxyprogesterone (DMPA, DepoProvera)?

◯ Yes
◯ No

6c1.) How are the injections given? (select all that apply)

☐ Subcutaneously
☐ Intramuscularly (gluteal)
☐ Intramuscularly (thigh)
☐ Intramuscularly (arm)
☐ Other

6c1.) Please specify: __________________________________

6d.) Do you prescribe implantable medications to be inserted subdermally, such as the etonogestrel or levonorgestrel contraceptive implants (Jadelle, SinoImplant, Implanon, or Nexplanon)?

◯ Yes
◯ No

7.) If a long-acting injectable formulation for malaria prevention was available for each of the following groups, how likely would you use it compared to the oral option?

	More likely than the oral option	Less likely than the oral option	Similar to the oral option
For adults:	◯	◯	◯
For adolescents (aged 12 and older):	◯	◯	◯
For children (aged 11 and younger):	◯	◯	◯

8.) Different long-acting injectable formulations require various frequencies of injections. Based on injection frequency, how likely would you be to use if injections were required...

	Definitely would try it	Might try it	Definitely would not try it
...once a month	○	○	○
...every two months	○	○	○
...every three months	○	○	○

9.) Please rank your preferred injection site for administration of a long-acting malaria prevention medication, from most preferred to least preferred injection site.

	1st preference	2nd preference	3rd preference	4th preference
IM deltoid	○	○	○	○
IM gluteal	○	○	○	○
IM thigh	○	○	○	○
Subcutaneous	○	○	○	○

10.) Please rank the following administration sites for of long-acting malaria prevention by injection, from most preferred to least preferred.

	1st preference	2nd preference	3rd preference
At home by the patient/family member after instructions/teaching has been provided	○	○	○
During visits to a clinical site	○	○	○
By a community healthcare worker in or near the patient's home	○	○	○

11.) Putting aside the route of administration, rate the following potential benefits for long-acting malaria prevention injectables using a 1-3 scale, where 1 is "not much benefit, and 3 is "very much a benefit":

	1-Not much benefit	2-Somewhat a benefit	3-Very much a benefit
Improved public health associated with long-term malaria prevention	○	○	○
Increased confirmation of patient adherence to a prescribed regimen	○	○	○
More effective option compared to oral therapy	○	○	○
Lower risk of adverse effects	○	○	○
Protection of healthcare privacy	○	○	○
Minimize stigma associated with taking treatment	○	○	○

12.) Putting aside the route of administration, rate the following potential barriers for a long-acting malaria prevention injectable using a 1-3 scale where 1 is "Not a barrier", and 3 is "Very much a barrier":

	1-Not a barrier	2-Somewhat a barrier	3-Very much a barrier
Higher cost of medication	○	○	○
Increased length of clinic visit	○	○	○
More frequent clinic visits	○	○	○
Concern about adverse effects	○	○	○
No benefit over already available oral options	○	○	○
Refrigeration requirement	○	○	○
Powder reconstitution prior to injection	○	○	○

13.) Any other comments (or reasoning if survey is incomplete)?"
